# Taste processing in *Drosophila* larvae

**DOI:** 10.3389/fnint.2015.00050

**Published:** 2015-10-13

**Authors:** Anthi A. Apostolopoulou, Anna Rist, Andreas S. Thum

**Affiliations:** ^1^Department of Biology, University of KonstanzKonstanz, Germany; ^2^Zukunftskolleg, University of KonstanzKonstanz, Germany

**Keywords:** *Drosophila* melanogaster, larvae, taste processing, gustation, single cell analysis

## Abstract

The sense of taste allows animals to detect chemical substances in their environment to initiate appropriate behaviors: to find food or a mate, to avoid hostile environments and predators. *Drosophila* larvae are a promising model organism to study gustation. Their simple nervous system triggers stereotypic behavioral responses, and the coding of taste can be studied by genetic tools at the single cell level. This review briefly summarizes recent progress on how taste information is sensed and processed by larval cephalic and pharyngeal sense organs. The focus lies on several studies, which revealed cellular and molecular mechanisms required to process sugar, salt, and bitter substances.

## Introduction

Gustation is one of the two major senses, along with olfaction, which enables animals to perceive their chemical environment. This applies to rather simple animals like the larvae of the fruit fly, too (Heimbeck et al., 1999; Colomb et al., 2007; Kwon et al., 2011; Stewart et al., 2015). *Drosophila* larvae highly depend on the food resources available at the site where they were placed as eggs. Therefore, gustatory information is of special importance to distinguish between edible, non-edible or even noxious substances (Heimbeck et al., 1999; Colomb et al., 2007; Kwon et al., 2011; Stewart et al., 2015).

Female *Drosophila melanogaster* flies lay their eggs on overripe fruits (Atkinson and Shorrocks, 1977). The embryonic development within the egg lasts about 24 h. After hatching, larval development takes about 5 days and includes three distinct instar stages defined by the molting of the larva. Finally, the larva pupates and undergoes metamorphosis into the adult fly, which takes about 5 days (Ashburner et al., 2005). Larvae spend most of their time foraging for food (Sokolowski, 1980; Green et al., 1983). Yeast that grows on the decaying fruits is their major source of proteins (Cooper, 1960; Becher et al., 2012), which are essential for development. Carbohydrates are important, too: larvae develop faster on a diet containing sucrose in addition to yeast (Schwarz et al., 2014). To recognize proteins and sugars but also salty and bitter substances, larvae need a sensory system that detects these substances and defines the preference or avoidance for them. This system is modifiable: in the mid 3rd instar, larvae switch from food-related to wandering behavior to select a food-free pupation site (Sokolowski et al., 1984).

In the last decades a number of studies have addressed the neuronal organization of the larval taste system. In addition, standardized assays have been established to assess taste driven behaviors (Python and Stocker, 2002; Colomb et al., 2007; Niewalda et al., 2008; Kwon et al., 2011; El-Keredy et al., 2012; Apostolopoulou et al., 2014; Stewart et al., 2015).

Compared to its adult counterpart, the larva displays a significantly simpler anatomical organization (Python and Stocker, 2002). Therefore, sensory neurons and receptor genes can be defined individually. Recently established genetic techniques allow to manipulate them. Thus, taste processing can be analyzed with cellular resolution on the anatomical, behavioral, molecular, and physiological level (Apostolopoulou et al., 2014). We suggest that the *Drosophila* larva is particularly suitable to study the mechanisms underlying the sensation and processing of taste.

## Neuronal fundamentals

### The larval chemosensory system

**Figure 1:**
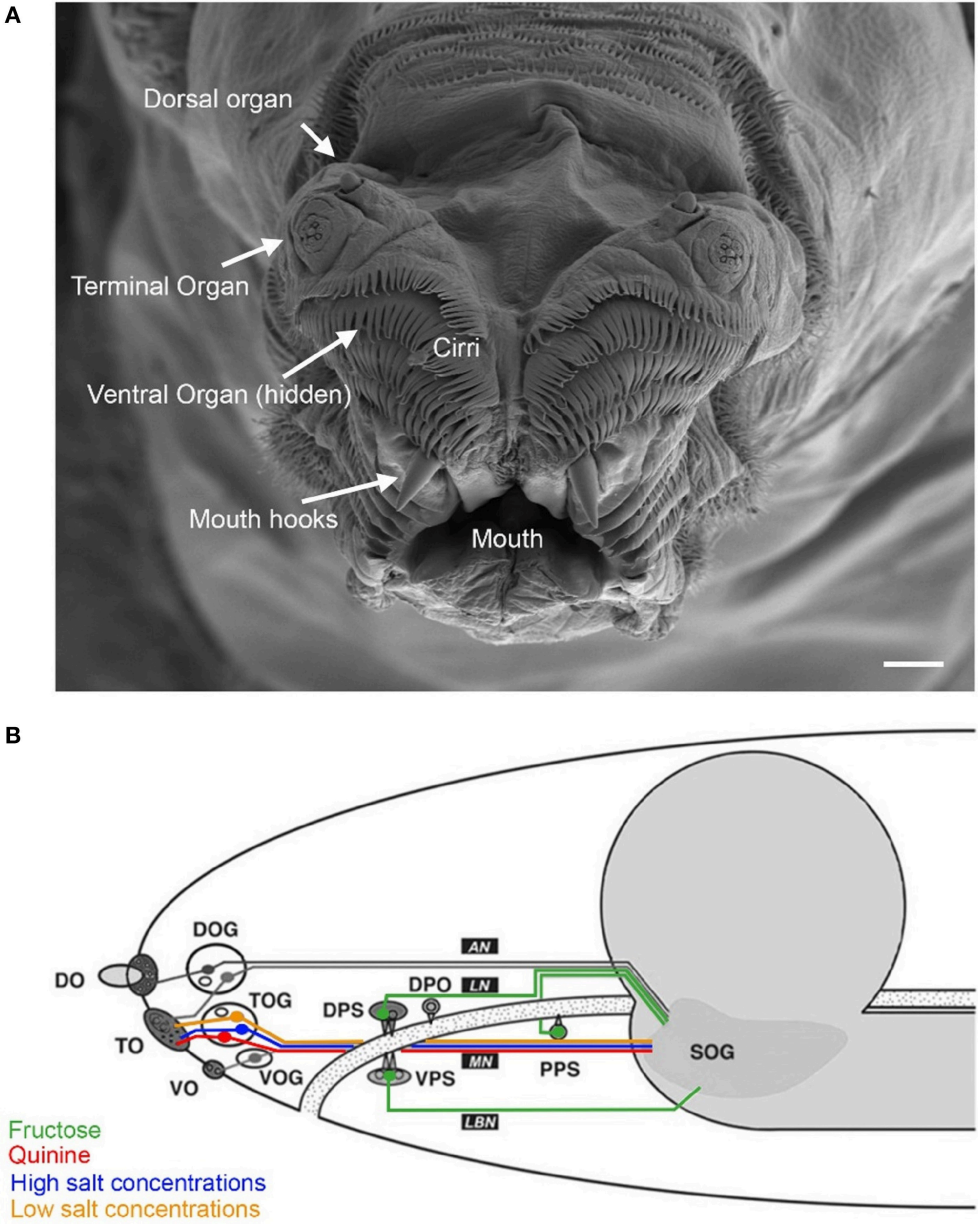
**Anatomy of the larval taste system**. **(A)** Fine structure of the larval cephalic organs: frontal view of the larval cephalon with the external sensory organs on each cephalic lobe: the dorsal organ (DO), the terminal organ (TO), the ventral organ (VO), which is hidden behind a row of cirri (hairlike, cuticle structures around the mouth opening). All organs are located as paired structures dorsally to the mouth and mouth hooks. **(B)** Schematic overview on the cephalic and pharyngeal chemosensory system. Shown are the major gustatory and olfactory organs, respective ganglions and central projections. Four main nerves connect the chemosensory organs with the central nervous system: antennal nerve (AN), labral nerve (LN), maxillary nerve (MN) and labial nerve (LBN). The brain is shown in gray. Olfactory processes of the DO innervate the larval antennal lobe via the AN. Putative gustatory DO projections are assumed to enter the subesophageal ganglion (SOG). Three cells from the DO ganglion send their dendrites into the TO. The TO and the VO project along the MN which enters the SOG. Four pharyngeal organs locate along the pharynx (PH). Projections from the VPS innervate the SOG over the LBN. The DPS, the DPO and the PPS send projections along the LN to the SOG. (Figure modified from Python and Stocker, 2002; Gerber and Stocker, 2007). Scale bar: 20 μm.

In larvae, at the peripheral sensory level, taste processing partially overlaps with the olfactory system. Therefore, we describe both sensory systems in combination (Figure 1). On the tip of the larval head three pairs of major external chemosensory organs are located: the dorsal (DO), the terminal (TO) and the ventral (VO) organ pairs (Figure 1A). In addition, four pairs of internal organs are located along the pharynx: the dorsal (DPS), the ventral (VPS) and the posterior (PPS) pharyngeal sensilla (Singh and Singh, 1984; Python and Stocker, 2002), and the dorsal pharyngeal organ (DPO) (Gendre et al., 2004) (Figure 1B).

The sensory organs' sensilla are innervated by bipolar neurons. Their cell bodies cluster in ganglia close to the respective organ. Their dendrite extends to the organs' surface and the single axonal projection ipsilaterally innervates the brain. Gustatory neurons of the cephalic and pharyngeal organs are supposed to terminate at the subesophageal ganglion (SOG), and olfactory receptor neurons at the larval antennal lobe (LAL) (Tissot et al., 1997; Python and Stocker, 2002; Kwon et al., 2011).

**Figure 2:**
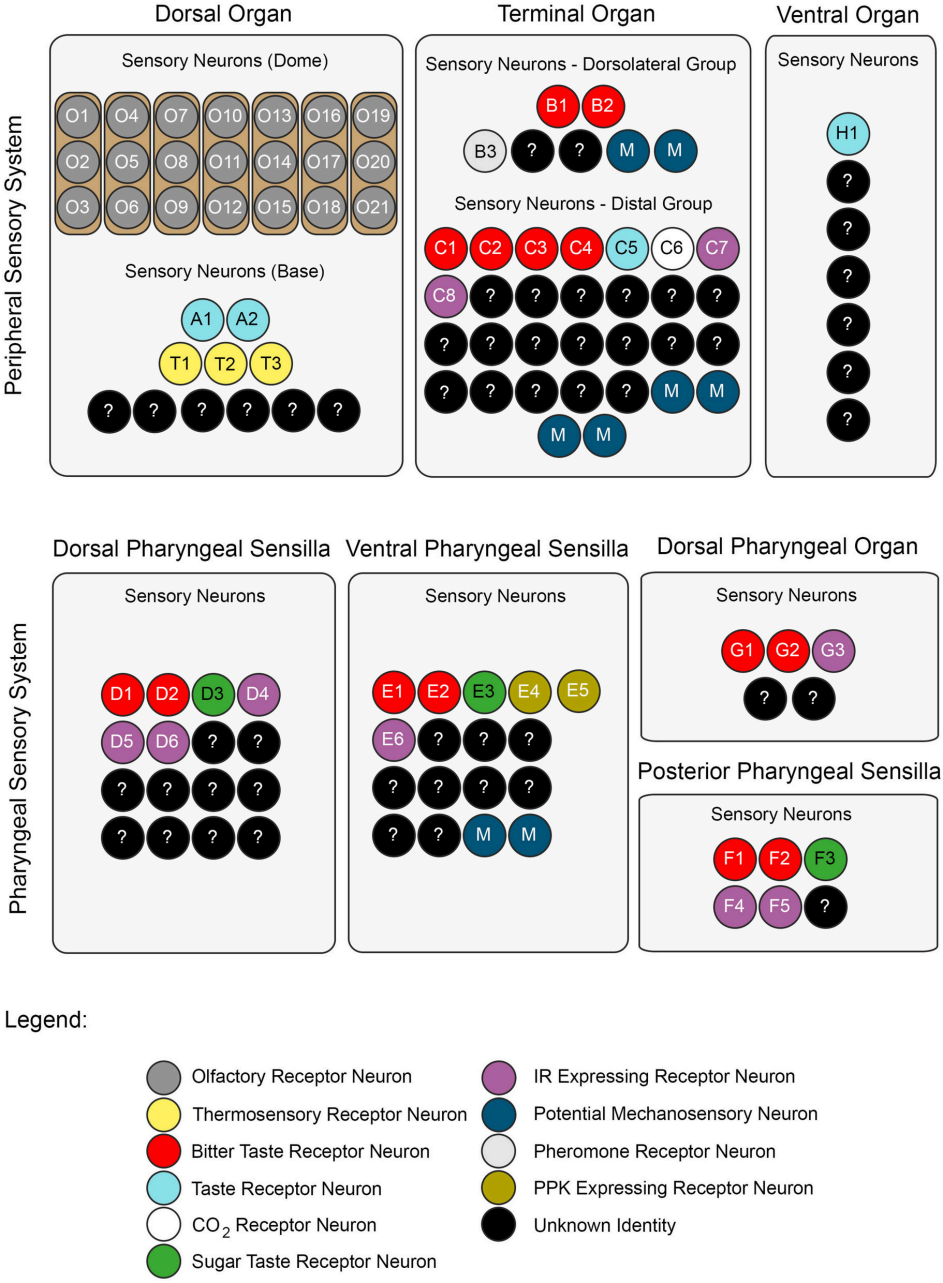
**A neuronal map of the larval taste system**. The neuronal map defines the single neurons of the larval taste system based on their molecular and functional properties. The peripheral chemosensory system of the larva consists of three major external organs. The DO dome comprises 21 olfactory sensory neurons, which all were shown to express ORs (Fishilevich et al., 2005; Kreher et al., 2005). There are additional 11 sensory neurons at its base (Sokolowski et al., 1984; Rohwedder et al., 2012). Of these, two neurons are putatively involved in taste sensing (A1 and A2) due to the expression of GRs (Kwon et al., 2011), and three further neurons mediate thermal stimuli (T1-T3) (Apostolopoulou et al., 2014). We consider the temperature sensitive neurons to be different than the GR-expressing ones, though this was not corroborated by co-localization experiments. The TO can be separated into a dorsolateral group and a distal group consisting of seven and 30 sensory neurons, respectively (Green et al., 1983; Sokolowski et al., 1984). The dorsolateral group was shown to perceive bitter taste (B1-B2) (Kwon et al., 2011), pheromone (B3) (Wang et al., 2004) and likely mechanosensory information (Green et al., 1983; Becher et al., 2012). The identity of two sensory neurons remains yet elusive. The distal group was suggested to mainly serve gustatory function. Its sensory neurons sense bitter as well as salt taste (C1-C4) (Niewalda et al., 2008; Mishra et al., 2013), taste (C5) and CO2 (C6) (Kwon et al., 2007, 2011). The function of further cells is unknown but they are characterized by the expression of IRs (C7 and C8) (Croset et al., 2010). In addition, some PPKs (PPK11, PPK6, PPK23) showed expression in neurons of the TO (Colomb et al., 2007). However, they were not mapped to defined neurons. Co-Expression with GRs is possible, because two PPK receptors, PPK12 and PPK23, were found in neurons that express GR66a, too (Colomb et al., 2007; Mast et al., 2014). However, the nature of the remaining neurons is unclear. The VO was often excluded from anatomical or functional studies (Sokolowski, 1980; Kwon et al., 2011). However, from ultrastructural data on house fly and fruit fly larvae gustatory function was derived (Sokolowski et al., 1984; Python and Stocker, 2002; Schwarz et al., 2014). Taste sensing is corroborated for at least one neuron due to the expression of a GR, GR2a, (H1) (Colomb et al., 2007). The pharyngeal sensory system of the larva consists of four sensory organs. The DPS provide mainly gustatory function. They house bitter (D1 and D2) (Kwon et al., 2011) and sugar sensing neurons (D3) (Mishra et al., 2013). Additionally, four IRs (IR60e, IR67c, IR60b, and IR94f) are expressed in three neurons (D4–D6) (Stewart et al., 2015). Hence, the identity of 10 additional sensory neurons is unknown. Expression of several other GRs was found in the DPS but not mapped to defined neurons (Kwon et al., 2011). The VPS was assumed to serve gustatory function, too. Bitter (E1 and E2) and sugar sensing neurons (E3) are corroborated. A PPK receptor, PPK6, is expressed in two neurons (E4 and E5) (Chu and Axtell, 1971; Colomb et al., 2007; Kwon et al., 2011), and one IR, IR11a, in one neuron (E6) (Croset et al., 2010). Their function is unknown. Because two more neurons were suggested to perceive mechanosensory input (Green et al., 1983; Sokolowski et al., 1984), the identity of additional nine neurons remains elusive. The DPO consists of only five sensory neurons (Gendre et al., 2004; Colomb et al., 2007). One neuron (G1) is labeled by the IR20a-Gal4 driver (Stewart et al., 2015). Two further neurons are putatively bitter sensing, due to the expression of GR66a (G2 and G3). Interestingly, one of them co-expressed PPK12 (Colomb et al., 2007). The PPS consists of six sensory neurons that probably serve gustatory function (Python and Stocker, 2002). Two neurons sense bitter (F1 and F2) and another one sweet (F3) (Dethier and Gelperin, 1967; Chu and Axtell, 1971). Two further neurons can be characterized due to the expression of IR100a (F4 and F6) (Croset et al., 2010). Seven additional GR-Gal4 lines label cells in the PPS (Kwon et al., 2011). In all taste organs, IR76b and IR25a showed expression in a broad number of cells and therefore were assumed to be co-receptors (Croset et al., 2010; Stewart et al., 2015). The identity of proposed neurons was not in every case definitely corroborated by co-expression studies. Nevertheless, we consider the “bitter” GRs, GR66a and GR33a to be co-expressed in bitter sensing neurons in all organs, though this was only shown for the TO (Kwon et al., 2011; Apostolopoulou et al., 2014). Further, we assume IR-expressing neurons to be different from GR- and PPK-expressing neurons, because they have not been shown to co-express with these receptor genes, yet. For some receptor genes, e.g., GR66a, the numbers of associated neurons varied in the literature (Colomb et al., 2007; Kwon et al., 2011). In this case, the lower number was chosen for the presented neuronal map. An exception was GR43a, of which we included the expression data from Mishra et al. (2013).

The DO can be divided into two substructures with distinct sensory functions: a multiporous dome-shaped compound sensillum encircled by additional six sensilla. The prominent “dome,” which has olfactory function, houses 21 olfactory receptor neurons organized in seven triplets. In total, eleven neurons innervate the six peripheral sensilla, which are, based on anatomical studies, assumed to mainly serve gustation (Chu and Axtell, 1971; Singh and Singh, 1984; Heimbeck et al., 1999; Oppliger et al., 2000; Python and Stocker, 2002; Fishilevich et al., 2005; Kreher et al., 2005). However, a recent study described three thermosensory neurons in the DO that probably innervate these sensilla (Figure 2) (Klein et al., 2015).

The cephalic TO and VO, and the four pharyngeal organs are the larval main gustatory organs (Python and Stocker, 2002). The TO comprises about 37 sensory neurons organized in 17 sensilla. Electrophysiological experiments corroborate its role in taste perception (Oppliger et al., 2000). However, ultrastructural studies indicate a more diverse function: the TO sensilla might also serve other modalities like mechano-, thermo- or hygrosensation (Chu-Wang and Axtell, 1972; Singh and Singh, 1984). The VO is located on the ventral side of the cephalic lobes and consists of seven neurons organized in five sensilla. Their morphology suggests a role in gustation and mechanosensation (Singh and Singh, 1984; Python and Stocker, 2002).

The pharyngeal sensory organs are organized in the following way: The DPS consists of about 16 neurons in six sensilla, the VPS of about 17 neurons in four sensilla, the DPO of only five neurons, and the PPS of six neurons organized in two sensilla (Singh and Singh, 1984; Python and Stocker, 2002; Gendre et al., 2004). Based on their anatomical properties a gustatory function was assumed (Singh and Singh, 1984; Python and Stocker, 2002; Gendre et al., 2004).

Taken together, the chemosensory system of the DO, TO, VO, DPS, VPS, DPO, and PPS consists of only about 119 sensory cells (Figure 2). As 21 of them have olfactory function, and at least additional 17 might serve other modalities, like mechano- or thermosensation, a maximal number of only 81 potential gustatory sensory neurons establishes the larval peripheral taste system (Python and Stocker, 2002). Therefore, gustatory information within the SOG relies on these few cells, too. However, multimodal functionality cannot be excluded for these sensory neurons. Accordingly, the above proposed numbers would be different.

It can be assumed that every single pair of sensory neurons has a unique response profile due to the expression of a single or several different types of receptors (Kwon et al., 2011; Stewart et al., 2015). Therefore, similar to the olfactory system, the larval taste system lacks cellular redundancy (Ramaekers et al., 2005).

## Molecular fundamentals

### Gustatory receptor genes (GR)

The gustatory receptor gene family (GR) in *Drosophila* consists of 68 members encoded by 60 GR genes (Clyne et al., 2000; Scott et al., 2001). They broadly serve in gustation: GRs were shown to detect sweet (Wang et al., 2004) and bitter (Weiss et al., 2011) stimuli in adults and larvae (Mishra et al., 2013; Apostolopoulou et al., 2014) but also non-volatile pheromones (Bray and Amrein, 2003), so far only corroborated for adult *Drosophila*. In addition, GR expression was observed in other types of sensory and central neurons (Thorne and Amrein, 2008), but also in endocrine cells of the gut (Park and Kwon, 2011). Therefore, they might have additional yet unidentified functions.

In larvae, GRs were described anatomically by studying the expression patterns of Gal4 lines (Colomb et al., 2007; Kwon et al., 2011). Kwon et al. (2011) found 43 GR-Gal4 lines which drove expression in larvae, 39 of them in the larval gustatory system. Each identified gustatory receptor neuron expressed a distinct set of multiple GRs and therefore allowed the authors to establish a receptor-to-neuron map for the DO and TO. Surprisingly, GRs covered only about one quarter of the larval gustatory system: they were expressed in 22 of the 81 potential gustatory neurons (Figure 2).

### Ionotropic receptor genes (IR)

Ionotropic receptors (IRs) are a family of recently identified chemosensory receptors in *Drosophila*. The IR family consists of 61 ionotropic glutamate receptors expressed in adult sensory neurons, which do not additionally express any ORs or GRs (Benton et al., 2009; Zhang et al., 2013). IRs might play a role in gustation and olfaction (Benton et al., 2009; Zhang et al., 2013).

In larvae, 14 members of the IR family were so far shown to be expressed in the gustatory organs throughout larval development but only 10 of them in 3rd instars (Croset et al., 2010; Stewart et al., 2015). Stewart et al. (2015) recently examined a subgroup of IRs, the IR20a clade, that includes about 35 members. The organization of the IR20a clade appears different from that of the GR or the OR family in larvae: IR-Gal4 lines did not label TO neuronal cells. Instead, eight of them drove expression in sensory neurons of the pharyngeal taste organs that varied between the different larval stages. Based on co-labeling experiments it was concluded that there are at least three distinguishable pairs of DPS neurons in 3rd instar larva each expressing a different member of the IR20a clade (Stewart et al., 2015). However, Croset et al. (2010) analyzed members of a different IR clade and found expression in the terminal organ for one line, IR7a-Gal4. In addition, two other lines labeled sensory neurons in the VPS and PPS respectively (Croset et al., 2010). Remarkable is the expression pattern of IR76b and IR25a, which in contrast to above listed IRs, were expressed in a broad number of sensory neurons of all taste organs. Therefore, they were assumed to function as co-receptors.

In summary, IRs seem to be expressed in at least nine neurons of the potential 81 bilateral larval gustatory neurons. For clarity reasons, the broadly expressed IR25a and IR76b, and IRs without expression in the 3rd instar were not included in the presented neuronal map (Figure 2) (Stewart et al., 2015).

So far, nothing is known about the functional contribution of IRs in larvae.

### Pickpocket genes (PPK)

The DEG/ENaC pickpocket receptor gene (PPK) family has been identified in many multicellular organisms across the animal kingdom. Individual ENaC subunits associate as homo or heteromultimers to form voltage insensitive, amiloride sensitive sodium channels. Their function seems to be very diverse (reviewed in Ben-Shahar, 2011). In *Drosophila*, 31 members of the pickpocket family were identified so far, each representing a channel subunit (Ben-Shahar, 2011). In larvae, previous studies showed that PPK1 is involved in nociception (Zhong et al., 2010), and that PPK11 might be involved in liquid clearance in trachea (Liu et al., 2003a). In addition, Mast et al. (2014) showed that larvae produce two long-chain fatty acids that are attractive to other larvae. These pheromone stimuli are detected by a single sensory neuron in each TO. On the molecular level PPK23 and PPK29 are required to respond to these pheromones (Mast et al., 2014).

Regarding gustatory function, PPK receptor genes might be involved in water sensation and salt taste. In adults, a study by Chen et al. (2010) revealed that PPK28 might serve as osmolarity sensor for gustatory water reception. In larvae, PPK subunits seem to contribute to salt taste: PPK11 and PPK19 were found to be expressed in gustatory organs. Disrupting these genes affected the larva's ability to discriminate low salt concentrations and affected the behavioral response to high salt concentrations (Liu et al., 2003b; Alves et al., 2014).

### Transient receptor potential channel (TRP)

TRP channels are cation channels, which are conserved throughout the animal phylogeny. They display remarkable diversity in their modes of action including sensory modalities like vision, thermosensation, olfaction, hearing and touch (Fowler and Montell, 2013; Venkatachalam et al., 2014). TRP channels seem to be the primary receptors for nociceptive compounds including menthol and capsaicin (Bandell et al., 2007). In addition, they have been shown to take part in gustatory sensing of acids (Huang et al., 2006). TRPs were also found to serve gustation. Two members of the TRP receptor gene family were found to be involved in gustation by mediating hygrosensation (Liu et al., 2007). In larvae, it was suggested that the TRP painless is required for fructose avoidance and migration to food-free sites before pupation. The related sensory neurons were assumed to be located at the thoracic segments (Xu et al., 2008). This finding indicates that additional sensory neurons might contribute to the larval gustatory system.

## Behavioral and functional fundamentals

### Sugar sensing

*Drosophila* larvae cover their metabolic needs in carbohydrates by consuming a mixture of fructose, glucose, sucrose and other sugars, which are available in fruits (Widdowson and McCance, 1935). In the laboratory, they can sense and do prefer different sugars in preference assays. These responses are dependent on concentration (Miyakawa, 1982; Schipanski et al., 2008; Rohwedder et al., 2012).

The neuronal and molecular background of sugar sensing in larvae has been puzzling: eight gustatory receptor genes (GR5a, GR61a, GR64a-f), which perceive different aspects of sugar information in adults (Dahanukar et al., 2007; Slone et al., 2007) showed no expression in the larvae (Colomb et al., 2007). But Mishra et al. (2013) proposed that the receptor gene GR43a is the main sugar receptor in larvae: GR43a was reported to be expressed in the brain, in the pharyngeal organs, as well as in neurons innervating the lumen of the larval foregut (Mishra et al., 2013).

Sweetness indicates the presence of sugars and caloric content. However, sweet taste can be an unreliable predictor of nutrient value because some sugars cannot be metabolized. Therefore, it is important for larvae to not only detect the taste, but in addition the nutritional value of the food to appropriately cover their metabolic needs. Actually, it was shown that in the presence of sugars with nutritional value (such as fructose, sucrose, glucose maltodextrin and sorbitol) larvae decrease their feeding behavior compared to that on pure agarose. On the contrary, in the presence of sugars without nutritional value (such as xylose and arabinose) feeding remains comparable to feeding on pure agarose (Rohwedder et al., 2012). In addition, larvae are able to perceive and to prefer sugars as rewarding independent of their nutritional value or their sweetness (Rohwedder et al., 2012). Thus, it is obvious that larvae can perceive different characteristics of sugars.

### Bitter sensing and processing

Bitter sensing is important for larvae in order to avoid noxious substances. Recently, a few studies have investigated larval bitter sensing of quinine, a substance perceived as bitter by humans. El-Keredy et al. (2012) have shown that larvae respond negatively to quinine: they avoid it, they feed less if it is included in a substrate, and perceive it as punishment during associative conditioning (El-Keredy et al., 2012). In adults, GR66a positive GRNs were identified as “bitter” neurons (Weiss et al., 2011). Similarly, in larvae co-expression of GR66a and GR33a was suggested to define a set of only 12 “bitter” neurons in total (six neurons in the TO, two in the DPS, VPS, and PPS, respectively) (Kwon et al., 2011). Indeed, neuronal activation of these was necessary to drive quinine dependent choice behavior and was sufficient to initiate aversion (Colomb et al., 2007; Apostolopoulou et al., 2014). Furthermore, single cell analysis revealed that the C3 neuron of the TO (besides the joined action of the C1, C2, and C4 neurons) mainly contributes to this response (Figure 2) (Apostolopoulou et al., 2014). Therefore, it was suggested that choice behavior is instructed by sensory neurons of the TO (Figure 1B).

### Salt sensing and processing

Sodium chloride is necessary for many physiological processes of animals. Therefore, it is important that larvae sense and precisely regulate its intake. In line with this, larvae prefer low and avoid high salt concentrations (Niewalda et al., 2008). Furthermore, larvae perceive the former as rewarding and the latter as punishing (Niewalda et al., 2008). The concentration dependent shift from appetitive to aversive perception depends on the diet of the larvae: They prefer salt concentrations lower than those consumed in their diet (Russell et al., 2011).

As mentioned above, it was observed that PPK11 and PPK19 (expressed in three and at least one neuron of the TO, respectively) are necessary for salt perception (Liu et al., 2003b; Alves et al., 2014). In addition, the serrano protein, assumed to be co-expressed with GR66a in the TO, was shown to be required for the detection of high salt concentrations (Alves et al., 2014). Thus, salt perception can be linked to the TO (Figure 1B).

### Amino acid sensing and processing

In nature, larvae cover their protein needs by feeding on yeast which grows on fruits (Becher et al., 2012). Recent data revealed that aspartic acid can be used as appetitive reinforcer to induce associative olfactory learning in larvae (Schleyer et al., 2015). In addition, larvae can detect a lack of essential amino acids in their food. Dopaminergic neurons sense amino acid imbalance through GC non-derepressing 2 (GCN2) kinase and GABA signaling, which induces avoidance of the deficient diet (Bjordal et al., 2014).

### Interaction of bitter-sweet and salt-sweet processing

Recently, Konig et al. (2015) revealed that sweet processing in larvae interacts with bitter and salt processing. In detail, they showed that quinine inhibits fructose dependent choice behavior. In addition, high salt concentrations inhibited glucose dependent choice behavior. Both in a concentration dependent manner. The 12 identified “bitter” neurons were not involved in quinine-induced fructose inhibition (Konig et al., 2015). Therefore, the neuronal and molecular background of these interactions remains to be investigated.

## A neuronal map of the larval gustatory system

As outlined in previous paragraphs, several studies collected information of the expression and function of different receptor gene families in the larval gustatory system. In Figure 2 we propose a neuronal map of the gustatory system, which defines associated neurons based on their molecular and functional properties. It shows that data is only available for a fraction of the potential taste neurons. Gustatory receptors are only represented sparsely. This finding is based on the expression pattern of Gal4 driver lines (Colomb et al., 2007; Kwon et al., 2011; Mishra et al., 2013), which might, due to technical restrictions, underestimate the endogenous expression of GRs. Nevertheless, the presence of other types of receptor genes is likely. Hence, which receptor genes might be expressed in the remaining “empty” taste neurons? Members of the IR, PPK and TRP gene families are without doubt promising candidates. However, their analysis added only a few more receptors to the larval taste system so far; the presence of TRPs was not reported at all. A reason therefore certainly is the restricted availability of Gal4 lines of the TRP and PPK family. In principal, it is questionable, if all of the proposed taste neurons (Python and Stocker, 2002) indeed exclusively serve the sensation of taste modalities. Likely, some of them instead serve hygro-, osmo-, proprio- or mechanosensation as indicated by ultrastructural properties of the sensilla described in house fly larvae. Elucidating, how and in which sensillum types sensory neurons are organized will be crucial to understand their functionality.

## Additional taste systems

Several studies were recently initiated to analyze taste processing in *Drosophila* larvae. The work exclusively focused on the role of the peripheral and the pharyngeal taste organs. These organs serve in evaluating the food quality. However, most likely there are additional checkpoints further downstream that collect sensory information to organize subsequent food dependent behaviors.

In fact, Park and Kwon (2011) showed that 15 GR-Gal4 drivers express in the gut of the adult. Although we miss such an analysis for larvae, different studies have shown that Gr-Gal4 lines express in the larval gut, too (Park and Kwon, 2011) (e.g., Gr43a-Gal4 shows expression in the proventriculus, Mishra et al., 2013). Therefore, the gastrointestinal tract might contribute to food information signaling.

Another system of importance is the stomatogastric system (Spiess et al., 2008). Early studies in the blowfly by Dethier and Gelperin (1967) have demonstrated that cutting the recurrent nerve, which connects the stomatogastric system with the brain, leads to hyperphagia. The authors' further showed that information from the foregut region, which controls feeding behavior, is lost by this treatment. The result was excessive food intake.

## Perspectives

A primary goal in neuroscience is to understand how animals detect, discriminate and respond to the huge variety of sensory stimuli in the environment. A simple nervous system, like that of *Drosophila* larvae, offers the possibility to study the neuronal correlates underlying these complex processes. Knowledge about their chemosensory system emerges rapidly. In order to enhance our understanding of the processing of taste in larvae, we suggest that future studies would benefit from clarifying the contribution of the stomatogastric and gastrointestinal systems to taste perception.

Furthermore, it is promising to advance the anatomical and functional dissection of the larval taste system on the single cell level. A deeper knowledge of the peripheral organization of taste organs and neurons will improve our understanding of their functionality. Therefore, the nature of the so far unidentified sensory neurons needs to be revealed by screening an expanded set of receptor gene Gal4 lines and subject them to a precise analysis for co-expression. However, due to technical restrictions Gal4 driver lines might not reflect the true endogenous expression pattern of sensory receptor genes. *In situ* hybridization would be preferable to corroborate receptor gene expression, but an effective protocol for larvae has not been established, yet.

### Conflict of interest statement

The authors declare that the research was conducted in the absence of any commercial or financial relationships that could be construed as a potential conflict of interest.
